# ‘I don’t really understand this BP’: Women’s knowledge, attitudes, and experiences with preeclampsia in Ghana

**DOI:** 10.1371/journal.pgph.0000121

**Published:** 2022-07-13

**Authors:** Avina Joshi, Titus K. Beyuo, Samuel A. Oppong, Cheryl A. Moyer, Emma R. Lawrence

**Affiliations:** 1 University of Massachusetts Medical School, Worcester, Massachusetts, United States of America; 2 University of Ghana School of Medicine and Dentistry, Accra, Ghana; 3 Department of Obstetrics & Gynaecology, Korle Bu Teaching Hospital, Accra, Ghana; 4 Global REACH, University of Michigan Medical School, Ann Arbor, Michigan, United States of America; 5 Department of Obstetrics & Gynecology, University of Michigan Medical School, Ann Arbor, Michigan, United States of America; University of California San Francisco, UNITED STATES

## Abstract

Preeclampsia and eclampsia are common and serious complications of pregnancies, often presenting as obstetric emergencies. In low- and middle-income countries, limited numbers of healthcare providers and a high volume of critically ill patients can negatively impact provider communication and counseling. Lack of knowledge or awareness of preeclampsia and eclampsia among pregnant women can lead to delays in health seeking behavior. Our study uses grounded theory to explore patients’ experience of preeclampsia and eclampsia in a low-resource setting. Participants were postpartum women diagnosed with preeclampsia or eclampsia at Korle Bu Teaching Hospital in Ghana. Interviews consisted of semi-structured, open-ended questions regarding participant understanding of their diagnosis of preeclampsia and eclampsia; counseling from their healthcare providers; and experiences with their delivery, monitoring, and treatment. Qualitative thematic analysis was performed according to the Attride-Sterling analytical framework, using NVivo 12. A total of 45 women were interviewed, 88.9% with preeclampsia and 11.1% with eclampsia. Major themes identified include participants’ low general knowledge of their diagnosis, inadequate counseling from healthcare providers, and resulting emotional distress. Women desire more information regarding their diagnosis and associate their health-seeking behaviors with counseling they receive from healthcare providers. Women also acknowledge the systemic barriers that make patient care and counseling challenging for providers, especially in low- and middle-income countries. These findings highlight the need for improved models of counseling and health education for women with pregnancies complicated by preeclampsia and eclampsia.

## Introduction

Preeclampsia and eclampsia are common and serious complications of pregnancies across the world [1, 2], affecting 2–10% of pregnancies [3] and contributing to maternal morbidity and mortality, as well as poor neonatal outcomes [4–6]. The incidence and burden of preeclampsia and eclampsia are higher in low- and middle-income countries (LMICs) like Ghana [1, 7]. In some hospitals in Ghana, preeclampsia and eclampsia have overtaken postpartum hemorrhage as the leading cause of maternal mortality [8, 9].

Preeclampsia and eclampsia are hypertensive disorders of pregnancy, categorized by new elevated blood pressures in pregnancy or postpartum. Clinically, preeclampsia is defined as blood pressure elevated to >140/90 on two separate occasions at least four hours apart, occurring after 20 weeks gestation in a patient with previously normal blood pressures, and associated with protein in the urine or other laboratory abnormalities or symptoms. Preeclampsia with severe features involves at least one of the following: blood pressure elevated to >160/110, physical symptoms (headache, vision changes, right upper abdominal pain) or specific laboratory abnormalities (platelets <100,000, liver enzymes elevated to twice normal, creatinine >1.1 or twice baseline) [10]. Eclampsia is the most serious manifestation of hypertensive disorders of pregnancy, and is defined by new seizures in pregnancy without another cause, such as an underlying seizure disorder or head trauma. The clinical presentation of preeclampsia can be either the development of symptoms outlined above, or newly measured elevated blood pressures. Eclampsia presents as seizures, which can be focal or generalized. If preeclampsia is not recognized and managed, patients are at risk of progression to eclampsia [10]. Patients with preeclampsia without severe features can be managed as an outpatient with very close monitoring of blood pressures, symptoms, and fetal status, with planned delivery at 37 weeks gestation. However, patients with preeclampsia with severe features or eclampsia require inpatient management. Treatment of preeclampsia with severe features and eclampsia centers on prompt delivery of the fetus and placenta, through either induction of labor or cesarean delivery. Additional treatment includes administration of antihypertensive medications to control blood pressures, injections of magnesium sulfate to reduce the risk of seizures, and monitoring for complications [10]. Both preeclampsia and eclampsia are associated with high risk of maternal complications, including acute kidney injury, cerebrovascular stroke, placental abruption, and maternal death. Neonatal complications are common and include preterm delivery, low birthweight, and stillbirth [10].

Preeclampsia and eclampsia often present as obstetric emergencies [7], which require prompt action [11], repeated administration of medications [12], and preterm delivery of neonates with Neonatal Intensive Care Unit (NICU) admission [4, 5]. Prolonged postpartum hospital admission is often needed for both mothers and their neonates [13]. In LMICs, the experience of labor and delivery can be stressful, overwhelming, and painful [14–16] due to obstetric environments being limited by the number of providers and a high volume of critically ill patients. This creates challenges for communication and counseling [17, 18].

While a significant amount of research has been conducted on the clinical care and healthcare outcomes of pregnancies complicated by preeclampsia and eclampsia, limited prior research has been done on the patient perspective. Our qualitative study aims to explore the experience of postpartum women in Ghana whose pregnancies were complicated by preeclampsia or eclampsia. Gaining a better understanding of attitudes, knowledge, and experiences of these patients may inform improved counseling and care of this high-risk obstetric population.

## Methodology

### Study setting and context

Our study was conducted at the Korle Bu Teaching Hospital, which is a large tertiary referral hospital located in Accra, Ghana. The maternity unit conducts approximately 10,000 deliveries per year. Patients receiving care at Korle Bu include those who reside in Accra, as well as referral cases throughout southern Ghana. In Ghana, the majority of women receive antenatal care through the government healthcare system, which consists of a hierarchical system of district hospitals, regional hospitals, and tertiary care hospitals. Although the World Health recommends at least eight antenatal visits in a pregnancy [19], only 40.7% of Ghanaian women meet this target [20]. Screening for blood pressure and urine protein is recommended at every antenatal visit to facilitate early diagnosis of preeclampsia [19]; data on rates of screening in Ghana is not available. At Korle Bu Teaching Hospital, consistent with standard recommendations as described in the introduction, patients with preeclampsia with severe features and eclampsia are managed with inpatient admission, administration of magnesium sulfate for seizure prophylaxis, antihypertensive medication, and prompt delivery [21–23].

### Participants

Study participants were pregnant patients admitted to the Korle Bu Teaching Hospital with a clinical diagnosis of preeclampsia with severe features or eclampsia. Inclusion criteria were age 18 years or older and fluency in either English or Twi/Akan.

### Recruitment

Participants were identified using admission logbooks on the maternity wards, which listed the name and basic demographic information for every admission to the maternity ward for labor or scheduled cesarean section. The logbooks and clinical charts of new admissions were reviewed every morning and evening by a research assistant. All participants with a qualifying diagnosis of preeclampsia or eclampsia were purposively recruited based upon their diagnosis. Recruitment and interviews were conducted until thematic saturation was reached, reflecting no new information being gained from subsequent interviews. No repeat interviews were performed.

### Data collection methods and tools

Grounded Theory, a qualitative approach for collecting and analyzing data without imposing previously constructed theoretical frameworks [23, 24], underpinned all aspects of this research. This approach was selected in order to capture participants’ perspectives without assuming they would conform to the researchers’ ideas about preeclampsia and eclampsia in Ghana. Open-ended discussions with women diagnosed with eclampsia and preeclampsia were used to generate a preliminary interview guide (rather than anchoring the interview guide in established health behavior frameworks). The preliminary interview guide was then pilot tested and refined to ensure it reflected women’s language and common understanding of preeclampsia/eclampsia.

The interview guide included probes focused on participants’ understanding of their diagnosis, experiences with counseling and treatment, challenges faced, and reflections on emotional and financial impact. The list of probes was written in English and translated to Twi/Akan by an external consultant with fluency in English and Twi/Akan and expertise in linguistics. Probes were then back-translated into English to ensure accuracy. Prior to data collection, the questions were pilot tested in a comparable population of pregnant women.

### Data collection approach

Data collection was completed between November 2019 and January 2020 by two trained research assistants, one of whom was an American female medical student and the other was a Ghanaian male research assistant fluent in Twi/Akan. Research assistant training focused heavily on qualitative interviewing techniques, using mock interviews, immediate feedback, and ongoing review and discussion of transcripts to ensure consistent and appropriate application of the principles of qualitative interviewing [25]. In addition, research assistant training included orientation to the sensitive nature of interview topics and culturally appropriate approaches to asking about adverse pregnancy outcomes.

Written informed consent was obtained from all participants. Participants had the option to pause or stop the interviews at any time, and to skip any questions that caused significant distress. Participants first met the research assistants during the informed consent process, and were told the job title and credentials of the research assistants. Research activities were conducted in either English or Twi/Akan, based on the participants’ choice.

Interviews were conducted face-to-face during the participants’ admission on the postpartum inpatient ward, at least two full days following delivery. The only people present for the interview were the participant and one research assistant. Each interview consisted of a series of semi-structured, open-ended questions regarding the patient’s perspective and experience of preeclampsia/eclampsia (see S1 File). The interview lasted approximately 30 to 60 minutes.

Demographic information was collected from each participant’s clinical record and entered into a REDCap database. Data included age, education level, clinical diagnosis (preeclampsia vs eclampsia), gestational age at delivery, mode of delivery, need for NICU admission, primary language used for healthcare, and length of hospital admission.

### Data management and analysis

Demographic data were transferred from REDCap to STATA (Version 16.0 StataCorp. 2) for statistical analysis. Interviews were audio recorded, those done in Twi/Akan were translated from Twi/Akan to English by a research assistant fluent in both languages, and then transcribed verbatim. The accuracy of translation was validated by review of five randomly selected transcripts by an external individual fluent in both English and Twi/Akan. No field notes were taken. Transcriptions were not returned to participants to review, and participants did not give feedback on the findings. Interview transcripts were entered into NVivo 12, which was used to organize the qualitative coding process. All transcripts were read by two of the researchers, who conducted independent in-vivo coding [26], and then worked together to generate a preliminary list of thematic codes. Codes were generated in keeping with tenets of grounded theory–allowing the data to speak for itself and not imposing a priori frameworks. A third researcher reviewed the list of codes against selected interviews. The research team then discussed each code and developed a coding dictionary. Each interview was coded according to the coding dictionary, with weekly discussions regarding any questions, concerns, or inconsistencies. Group discussions were used to resolve coding issues. Once the first round of coding was complete, the research team turned to the Attride-Sterling framework for thematic network analysis [27] to assist in better understanding how basic themes in the data might cluster into organizing themes and overarching global themes.

This process resulted in the final themes presented here. Reporting for this study was completed based on the COREQ checklist for qualitative research (see S1 Checklist) [28].

### Ethical considerations

Ethical approval for the study was granted by the Scientific and Technical Committee of the Korle Bu Teaching Hospital (KBTH-IRB 00096/2018) and the University of Michigan Institutional Review Board (HUM00139104).

## Results

Table 1 demonstrates the demographics of interviewed participants. Forty-seven sequential women meeting inclusion criteria were recruited and all agreed to participate, however two were discharged prior to the planned interview. A total of 45 participants were interviewed—88.9% (n = 40) with preeclampsia and 11.1% (n = 5) with eclampsia. Approximately half of the interviews (n = 24, 53.3%) were conducted in English and the remainder were conducted in Twi/Akan. Participants had a median age of 31 years and median parity of two. Forty-four percent (n = 20) of participants were primiparous. The median gestational age at delivery was 37.6 weeks, two-thirds of deliveries were via cesarean section (n = 30, 66.7%), and the median length of maternal hospital admission was seven days. Seven participants (15.6%) experienced a stillbirth. Of participants with a live birth, half of their babies required a NICU admission, with a median NICU admission of eight days.

**Table 1:** Demographic factors of participants (N = 45).

Characteristic	n (%) or median (range)
Language Used for Interview	
English	24 (53.3)
Not English	21 (46.7)
Age, years	31 (18–42)
Highest Level of Completed Education^a^	
None	1 (2.3)
Primary	16 (37.2)
Secondary	12 (27.9)
Tertiary	14 (32.6)
Clinical Diagnosis	
Preeclampsia	40 (88.9)
Eclampsia	5 (11.1)
Length of Hospital Admission, days	7 (3–30)
Parity	2 (1–6)
Primiparous	20 (44.4)
Multiparous	25 (55.6)
Gestational Age at Delivery, weeks	37.6 (21.3–42.3)
Mode of Delivery	
Vaginal	15 (33.3)
C-section	30 (66.7)
Outcome of Delivery	
Livebirth	38 (84.4)
Stillbirth	7 (15.6)
NICU Admission^b^	19 (50.0)
Length of NICU Admission, days	8 (1–60)
Status of Baby at Discharge^c^	
Alive	35 (92.1)
Dead	3 (7.9)

^a^Out of 44 participants due to missing data

^b^Out of 38 live births

^c^Out of 38 live births

**Fig 1:**
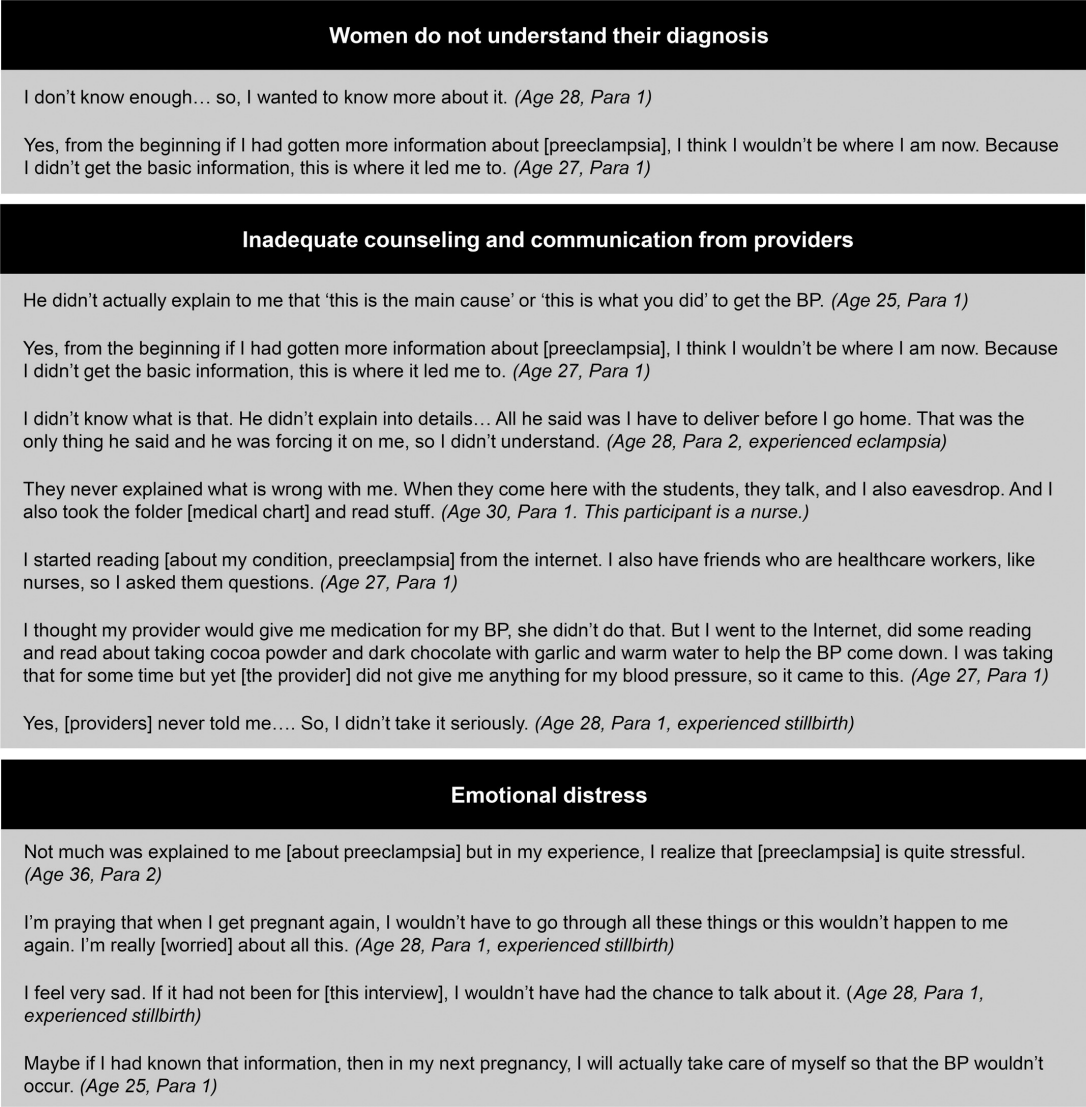
Representative quotations illustrating global themes.

The global themes identified include: 1) women do not feel confident in their understanding of their diagnosis; 2) women perceive inadequate counseling and education from healthcare providers; 3) both of these ideas contribute to significant emotional distress of women diagnosed with preeclampsia/eclampsia; and 4) the resulting negative emotions and lack of information can lead to problems with medical compliance among this population. The following sections provide illustrative quotes demonstrating each of these themes. Each quote is followed by the participant’s age and number of obstetric deliveries preceded by the term ‘para.’ If a participant’s diagnosis progressed from preeclampsia to eclampsia, it is noted below following her age and number of deliveries. We have also noted participants who experienced a stillbirth. If not directly specified, participants were diagnosed with preeclampsia and had a live birth. Additional quotes per theme can be found in Fig 1.

### Women do not understand their diagnosis

When asked about their knowledge of their diagnosis of preeclampsia or eclampsia, many participants responded that they did not know anything about the condition in general.

So far, I don’t know anything about it [preeclampsia]. I’ve never heard anything about this thing before… So… I don’t know about it. I don’t know what to say. (*Age 26*, *Para 1*, *experienced eclampsia*)

In fact, many participants did not even know the actual name of their diagnosis, and simply referred to it as ‘BP.’ This was consistent throughout many of the interviews.

I don’t actually understand this BP. (*Age 24*, *Para 2*, *experienced eclampsia*)

**Fig 2:**
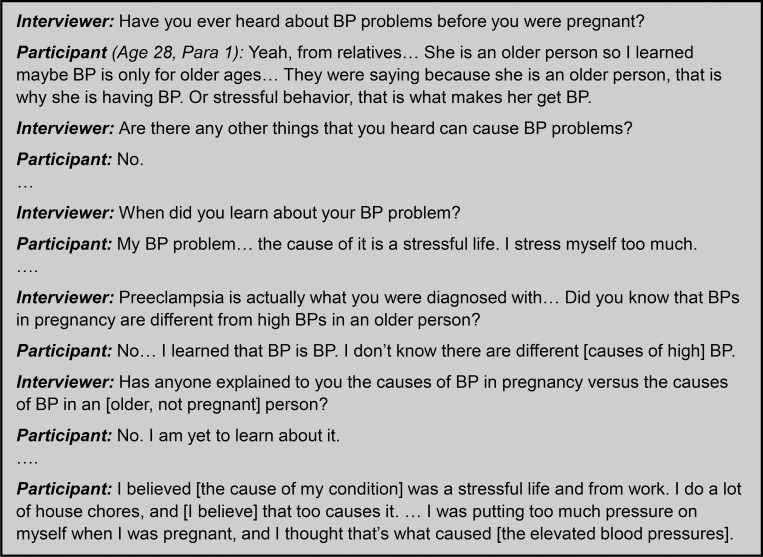
Interview excerpt demonstrating diagnostic confusion.

Referring to preeclampsia/eclampsia simply as ‘BP’ led to significant diagnostic confusion, as demonstrated by Fig 2. Participants were not able to distinguish their diagnosis from chronic hypertension, leading to confusion about both the diagnosis and the causes of the condition. Many participants incorrectly believed that emotional stress contributed to the development of their condition.

While many participants acknowledged they did not understand their own diagnosis, they expressed a desire to learn more about their condition.

I want more information so I can take good care of myself again in my next pregnancy. (*Age 39*, *Para 1*, *experienced eclampsia and stillbirth*)

Many participants specifically expressed a desire to know more information so that they could be more informed and take action in subsequent pregnancies.

[Providers] should explain to us what we did on our part to cause this so we can do something to prevent this from happening. *(Age 24*, *Para 2*, *experienced eclampsia)*

### Inadequate counseling and communication from healthcare providers

The majority of participants discussed at length the inadequacy of the counseling they received about their diagnosis from healthcare providers. Participants describe a healthcare setting that does not include the patient in discussion of her diagnosis.

They haven’t really explained anything to me. All they come and ask how I’m doing, go and do labs, get this medicine and [tell me to] take it. They never explained what is wrong with me. (*Age 30*, *Para 1 –this participant is a nurse)*

Inadequate explanations from providers led to dissatisfaction with medical care. Additionally, many participants described a general sense of dismissal regarding the patient’s input in their own healthcare.

I actually blame the place I was going for antenatal care because the nurses don’t let you explain, they don’t let you tell your side of it. They don’t actually ask you what is wrong with you for you to explain… Then they tell you “oh I guess you are fine because you have strength” [you look healthy]. (*Age 28*, *Para 1*, *experienced stillbirth*)

If participants wanted information, instead of relying on counseling from providers at antenatal visits, they had to have some baseline level of working knowledge of the condition—especially in knowing which questions to ask.

When I was going for antenatal, it seems that all the information that you need, you will have to ask. They don’t really give you much information… So, you have to ask virtually everything. (*Age 21*, *Para 1*)

In addition to the lack of disease-specific counseling, participants described a lack of explanations of procedures involved with preeclampsia treatment. Participants also spoke about being overwhelmed by the number of providers working on them at the same time.

One person should come one at a time. Because if I’m taking an [intravenous access] and they’re setting a line for me, I don’t expect you to be pricking my hand as well or injecting me, on the other side. Another person injected my buttocks here, no, it’s too much for me. I can’t take it. (*Age 30*, *Para 3*)

Participants expressed frustration about inadequate explanations, or explanations that were difficult to understand due to use of medical jargon. This led to significant negative feelings toward providers and how they treat admitted patients.

They treat us like we don’t know what’s going on…. [providers] just come and do whatever they like… and they will respond to you if they see fit… you can go to [a provider] and question them and nobody explains. (*Age 27*, *Para 1*)

I don’t think [providers give explanations to patients]. They just give you the term and then they just leave you to try and understand it on your own. And it’s not fair. (*Age 27*, *Para 1*)

Inadequate counseling and communication from healthcare providers led to participants seeking information via alternate sources (see Fig 1 for more examples).

That [A negative interaction where the physician failed to provide an explanation] was the reason I decided not to go to the hospital anymore. I decided to see an herbalist and she started to give me the BP drugs. (*Age 28*, *Para 2*, *experienced eclampsia*)

In some cases, a lack of counseling led to decreased compliance with medical advice.

Yes, I did not get a good explanation. So, the [healthcare providers] started to tell me to go and do something and I didn’t do it…. Will you blame me for that? (*Age 35*, *Para 4)*

### Emotional distress

Interviewed participants described many reasons why their experience with preeclampsia was challenging. These reasons included needing to undergo a caesarean section for delivery, the experience of medication administration, worrying about the well-being of their babies—especially those who were admitted to the NICU, and the financial burden caused by the condition. Additionally, it appeared the emotional distress that was experienced by many participants was related directly to their lack of understanding about the condition, to which their experience with providers was a major contributor.

No one told me anything and then just yesterday they told me I have this condition. I feel terrified and it’s painful. (*Age 28*, *Para 1*, *experienced stillbirth)*

Many participants described a sense of shock and feeling unprepared for preeclampsia/eclampsia and its consequences during their pregnancy. One respondent even commented directly that more information earlier in her pregnancy would have helped her cope with the condition.

All I can say is that if I had information earlier, then psychologically, I would have felt better. (*Age 30*, *Para 3)*

The negative emotions and lack of understanding of their condition also led to participants feeling responsible for development of the condition, resulting in feelings of self-blame.

It was quite stressful… I kept asking myself lots of questions. I wanted to know where I went wrong because I thought everything had been quite OK. (*Age 30*, *Para 3)*

The lack of information from providers contributed significantly to negative emotions and fear experienced by participants when they were diagnosed with preeclampsia. However, when participants did receive counseling about their diagnosis, they described some of their emotional burden being alleviated.

It was not easy because I was afraid [of the diagnosis of preeclampsia]… but they told me and they educated me about [preeclampsia]. They explained things to me and then I accepted it. (*Age 32*, *Para 4)*

### Provider barriers

**Fig 3:**
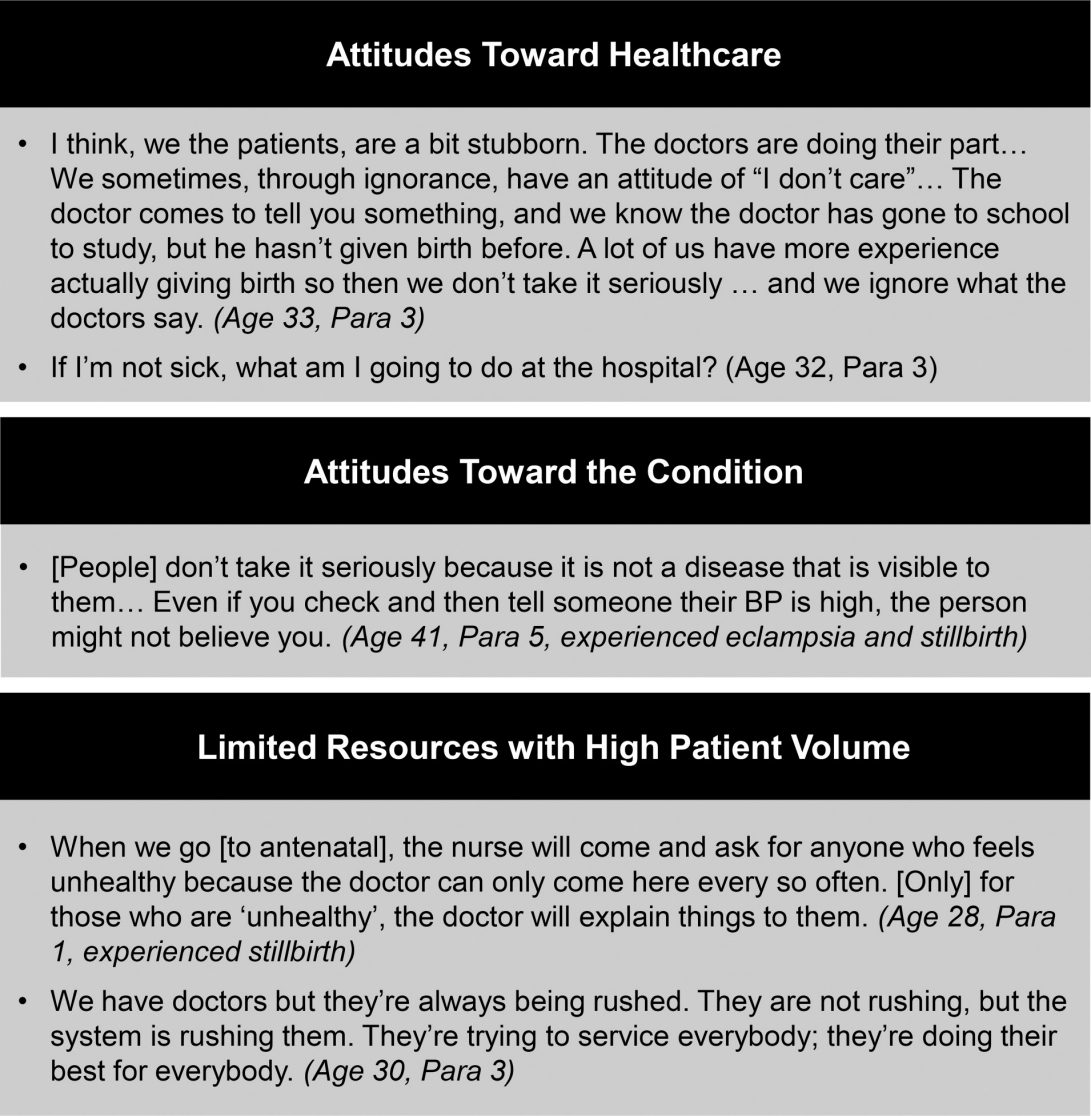
Barriers faced by health care providers.

While there is overwhelming evidence regarding inadequate communication, many participants explicitly recognized barriers that make it more challenging for providers to provide sufficient counseling (See Fig 3). Many participants acknowledged that providers have a very large number of patients to simultaneously care for.

[The providers] are doing their best. But … they have limitations. They don’t have enough time. They have so many people to attend to and I think their duties are quite huge. (*Age 30*, *Para 3*)

In addition, participants recognized provider limitations due to general attitudes and misperceptions toward the necessity of healthcare.

I thought [going to antenatal care] was not needed at all. *(Age 38*, *Para 3)*

These barriers, along with others acknowledged by participants, demonstrate just a few of the systemic barriers providers face when providing care and counseling to patients.

## Discussion

This qualitative study of 45 women with preeclampsia/eclampsia in Ghana illustrated that across age, education, and language spoken, there was a general lack of knowledge regarding the diagnosis, inadequate counseling by healthcare providers, and resulting significant emotional distress. Many women reported not knowing the name of their diagnosis or understanding what it was, and in some cases, those misunderstandings or diagnostic confusion directly contributed towards feelings of self-blame. Women also reported that providers did not include them in discussions about their own healthcare. Many reported that their questions were either not invited or were dismissed. This led to feelings of dissatisfaction with the medical system and frustration with medical care.

Many women expressed a desire to learn more about their condition and felt that being more knowledgeable would have allowed them to take action in their current pregnancy, or to make informed decisions in subsequent pregnancies. Some women even sought information from alternative sources; during antenatal care, they did this by talking with family and friends and getting advice from religious sources and traditional healers. During their inpatient admission, some participants reported reading their own medical chart and searching on the internet. It is worthy to note that participants attributed inadequate counseling from medical providers to their own non-compliance with medical recommendations. These themes were consistent across participants, including those who were fluent in English and those with high levels of education.

Limited prior research has been conducted on the patient perspective on preeclampsia, with the majority of existing literature focused on preeclampsia knowledge. The findings of this study are consistent with studies completed in both high-income countries, such as the U.S. and Australia [29–31], as well as LMICs, like Ghana, Nigeria, Tanzania, and Malaysia [32–37], which demonstrate overall low levels of knowledge regarding preeclampsia. This lack of knowledge is not only shown with objective knowledge assessments, but is also directly acknowledged by study participants themselves. While many of our participants expressed low levels of knowledge about preeclampsia, they also demonstrated a desire to have more information regarding their condition—whether through reading their own charts or seeking information on the internet or from friends. This is consistent with other studies that have demonstrated patient desire for more knowledge regarding preeclampsia [29]. This presents an opportunity for patient education, especially within the healthcare setting. In fact, several studies show patients have more objective knowledge regarding preeclampsia/eclampsia if they have had a discussion about their condition with a healthcare provider [31, 34, 38]. The lack of patient knowledge is tied to the nature of interactions with healthcare providers, as demonstrated by our participants who expressed frustration regarding the amount of initiative patients must take in order to ask ‘the right questions.’ This was also seen in a Nigerian study, which directly correlated the educational value of antenatal care visits with patient knowledge and directed questioning [36]. Without preexisting knowledge, patients are unable to gain more information from their providers. This has significant implications, as it is known that when women receive prenatal education on symptoms of preeclampsia, it may result in improved outcomes [39–43].

Overall, our participants expressed a general dissatisfaction with their interactions with healthcare providers. This is consistent with a wide body of literature worldwide regarding healthcare provider communication and the lack thereof, as well as maltreatment of women during maternity care [16, 36, 44, 45]. This maltreatment and lack of communication is even acknowledged by healthcare providers [16] and has a known detrimental effect on patient compliance and trust in their providers [44, 45]. In the long term, the quality of patient-provider interactions has the ability to strongly influence future health-seeking behaviors [36].

Prior studies on the emotional burden of preeclampsia and eclampsia have demonstrated women feeling scared, unprepared, guilty, and with a high sense of self-blame regarding their diagnosis of preeclampsia [30, 46], all of which are consistent with the sentiments expressed by our study participants. These studies have shown a direct link in lack of knowledge to those feelings of self-blame, as many women do not know the diagnostic causes of preeclampsia and often feel there was something they should have done better or differently to prevent it. Many women incorrectly believe changing their diet or reducing emotional stress could have prevented preeclampsia, leading to significant feelings of self-blame [46].

The lack of knowledge compounded by negative interactions with healthcare providers contribute to the emotional state of this already vulnerable population. Consistent with a few of our interviews, one study found that having a trusting and continuous relationship with their healthcare provider and feeling informed about their condition positively impacts a woman’s experience with preeclampsia [30]. While our data acknowledges there are many systemic barriers for healthcare providers (Fig 3), this presents an opportunity for increased education of preeclampsia to the general population. As suggested by our participants, this may imply more educational material provided in other forms, such as through television, the internet, or social media.

Ultimately, it is important to acknowledge the emotional impact of preeclampsia/eclampsia, especially due to the fact that many women feel a sense of unpreparedness and self-blame. As demonstrated by our data and several other studies, these feelings may be positively modified by informative interactions with healthcare providers to promote patient knowledge, empowerment, and agency. This further has the ability to impact life-long patient health-seeking behaviors.

Our study fills an important gap in the literature by exploring the patient perspective on preeclampsia and eclampsia in a low-resource setting. Nonetheless, this study has several limitations. First, our study was conducted at a single tertiary hospital in Ghana. While our qualitative study design precludes traditional discussion of generalizability, the Korle Bu Teaching Hospital provides care for women from urban Accra, as well as referrals from peri-urban and rural areas throughout southern Ghana. Participants represented a wide range of ages, parity, education level, and primary language of healthcare conversations, which supports the diversity of responses that were included. Second, interviews were conducted in an inpatient hospital setting, which may have made participants feel uncomfortable sharing responses that were critical of their healthcare providers or facility. To limit discomfort and promote honest responses, interviews were conducted by research assistants not connected with the healthcare team and the informed consent process included reassurance that participation would have no impact on their medical care. Third, there is not a direct translation of the clinical term “preeclampsia” or “eclampsia” in Twi/Akan, which is a primary local language spoken by many participants in our study. This may contribute to challenges for providers in delivering clear counseling, and to women’s lack of understanding of their condition. However, similar themes were seen across all participants, including those whose healthcare counseling and study interview was conducted in English.

## Conclusion

We demonstrate that women with preeclampsia and eclampsia experience common themes of low knowledge about their diagnosis, inadequate counseling by healthcare providers, and emotional distress. Women recognize barriers to communication with their providers, including high patient volume and limited time and number of providers. Women want to be more informed about their diagnosis, and some attribute their non-compliance with medical recommendations to the quality of counseling they receive. This connection is especially important, because occurrence of preeclampsia or eclampsia is a risk factor for recurrence in future pregnancies and negative experiences may impact future health-seeking behaviors. These findings highlight the need for improved models of counseling and health education that support patients, particularly those with recurring pregnancy complications like preeclampsia and eclampsia. Women’s acknowledgement of provider barriers also suggests that future studies on the provider perspective of counseling could be helpful in creating and implementing improved models of counseling and health education. Understanding the patient perspective on preeclampsia and eclampsia helps inform the patient’s understanding of their diagnosis, their experience with obstetric care, and their attitudes toward future engagement in the healthcare system.

## Supporting information

S1 Checklist COREQ checklist. (PDF)

S1 File Interview guide. (DOCX)
