# Supplementary material for: ‘I don’t really understand this BP’: Women’s knowledge, attitudes, and experiences with preeclampsia in Ghana
Source: PLOS Glob Public Health. 2022 Jul 13;2(7):e0000121. doi: 10.1371/journal.pgph.0000121 (PMC10022332; doi:10.1371/journal.pgph.0000121)
Supplement: S1 File — (DOCX) [file pgph.0000121.s002.docx]

**INTERVIEW GUIDE**

**Interview Guide/Script**

**“***Hello again. My name is ___________ and I am working on a research project with Dr. Beyuo here at Korle Bu. As we discussed, we are doing a research study on conditions of pregnancy and hope to know the patient perspective of care. We believe hearing from you will provide valuable information for our study and help us figure out ways to improve care for other women like yourself. As a thank you, after the survey, we will provide you with a small token to show our gratitude. It will take about 30-45 minutes to complete. I will be recording your responses to the open-ended questions. Is this something you feel comfortable doing right now?”*

If no, thank the patient for her time.
If the patient asks you to come back another time, plan a time that you will be able to return. **Avoid times when there will be visitors on the ward (Morning: 7-8AM, Afternoon: 4:30-5:30PM).**

If yes, continue with the following:

“*Thank you for agreeing. There are a few things I will go over with you before starting the interview. First and foremost, this interview is completely confidential. This means I cannot tell anyone about your answers. In addition, your responses will be de-identified and your name will not be associated with your responses. This applies to both the survey answers as well as the recorded answers in the open-ended portion. Second, I am not part of your health care team. The answers you give will not influence any current or future healthcare you are receiving, so feel free to be honest and open about your experience, as there will be no consequences. Third, if there are any questions that make you uncomfortable or you wish to stop the interview, you can let me know at any point. And lastly, feel free to ask me any clarifying questions if necessary. Before we begin, do you have any questions?”*

Answer any questions as needed.

*“If you have no other questions, then why don’t we begin. Please answer the questions as honestly as you can – there are no right or wrong answers. I want to know how you feel about your care, so you do not need to worry about giving me answers that you think I want to hear.”*

*I will be using a recorder as I talked about earlier. Again, the recorder is simply to make sure we get your entire response but your name will not be attached to the recordings.*

*During the surveys we were asking for short responses to multiple choice questions. However, for these next few questions, we want you to tell us more about your experience from your perspective. If you need some time to think about your responses, feel free to do so and answer how you best see fit. The more detail in these responses, the better, as it gives us a better understanding of your individual experience with preeclampsia. Do you have any questions about that?”*

Answer any necessary questions. Turn on the voice recorder and make sure it is recording.

1. How did the condition (preeclampsia or eclampsia) affect your just ended pregnancy?
2. What treatments did you receive?
   - 1. Do you know of any alternative treatments? If so, what are they?
3. Which aspect of the treatment was most troubling/difficult for you?
4. What aspect of your management does wish could be improved?
5. What caused preeclampsia or eclampsia your just ended pregnancy?
   - 1. Do you think it resulted from something you did or did not do?
6. Can preeclampsia or eclampsia be prevented?
   - 1. How?
7. What do you understand to be possible effects that this condition can have on the pregnancy (baby /mother)?
8. Do you know if the condition(s) you developed in this pregnancy has any future health consequences?

Notes for the Interviewer:

- The numbered questions serve as the **prompts** for the patients. Try to ask all prompts in an **open-ended** manner (avoid asking questions in a way that elicits a yes or no response). The bullet points serve as guide points for the interviewer for what questions can be answered within the prompt.
- If a patient answers a question with a yes or no response, probe further by asking her why that was her response.
- Allow the patient time to think through her responses. This may involve a lot of silence after each question.
- Follow the interview guide loosely – if the patient is speaking in depth about a certain topic, there is no rush to move on to the next topic until she has completed her thoughts.

**CLOSING**

“*We have now completed both sets of surveys and the open-ended interview portion. I thank you so much for your time and for participating in the survey. I want to reiterate that your answers were very valuable for us as we’re hoping to learn how we can improve patient care for you and women like you. Is there anything else you have questions on? Any other comments for the research team?”*

Address any questions or comments as necessary.

Present the patient with her incentive and thank her again for her time.
